# Near-wall hemodynamic parameters quantification in in vitro intracranial aneurysms with 7 T PC-MRI

**DOI:** 10.1007/s10334-023-01082-2

**Published:** 2023-04-19

**Authors:** Antoine Sache, Philippe Reymond, Olivier Brina, Bernd Jung, Mohamed Farhat, Maria Isabel Vargas

**Affiliations:** 1 Department of Mechanical Engineering, Ecole Polytechnique Fédérale de Lausanne, Lausanne, Switzerland; 2 Division of Neuroradiology, Geneva University Hospital, University of Geneva, Geneva, Switzerland; 3 Department of Diagnostic, Interventional and Paediatric Radiology, Inselspital, Bern University Hospital, University of Bern, Bern, Switzerland

**Keywords:** Intracranial aneurysms, 7 T phase contrast MRI, Hemodynamics, Wall shear stress, In vitro models

## Abstract

**Objective:**

Wall shear stress (WSS) and its derived spatiotemporal parameters have proven to play a major role on intracranial aneurysms (IAs) growth and rupture. This study aims to demonstrate how ultra-high field (UHF) 7 T phase contrast magnetic resonance imaging (PC-MRI) coupled with advanced image acceleration techniques allows a highly resolved visualization of near-wall hemodynamic parameters patterns in in vitro IAs, paving the way for more robust risk assessment of their growth and rupture.

**Materials and methods:**

We performed pulsatile flow measurements inside three in vitro models of patient-specific IAs using 7 T PC-MRI. To this end, we built an MRI-compatible test bench, which faithfully reproduced a typical physiological intracranial flow rate in the models.

**Results:**

The ultra-high field 7 T images revealed WSS patterns with high spatiotemporal resolution. Interestingly, the high oscillatory shear index values were found in the core of low WSS vortical structures and in flow stream intersecting regions. In contrast, maxima of WSS occurred around the impinging jet sites.

**Conclusions:**

We showed that the elevated signal-to-noise ratio arising from 7 T PC-MRI enabled to resolve high and low WSS patterns with a high degree of detail.

## Introduction

Intracranial aneurysms (IAs) are characterized by an abnormal localized dilatation of a cerebral artery resulting in ballooning. They impact roughly 3% of the population [1] and their rupture causing subarachnoid hemorrhage (SAH) carries a mortality risk of around 30–40% [2]. However, most aneurysms remain asymptomatic [3], and thus predicting their evolution with time is of a great interest. Apart from the classical morphological features (size, location, wall irregularity), it is widely believed that hemodynamics parameters, particularly wall shear stress (WSS) are directly related to IAs initiation, growth and rupture [4–6]. Therefore, their quantification deduced from knowledge of the blood velocity field, is necessary in clinics to prevent disastrous consequences for patients.

Wall shear stress, defined as the tangential stress exerted by the flowing blood on the vessel wall, is a known stimulus for arterial mechanotransduction, which can impact endothelial cells (ECs) function and result in vascular remodeling [7]. While high WSS has been demonstrated to initiate aneurysms formation [8–10], low WSS regions have been associated with aneurysm growth and rupture through arterial wall remodeling [3]. However, the impact of WSS is more subtle due to the heterogeneity of aneurysms size and morphology [11] and the possible patterns that could lead to symptoms [12]. In fact, there is still an important controversy about the effect of WSS, as both low and high WSS have been separately correlated with IAs growth and rupture [13]. To address both hypothesis, Cebral et al. [14] and Meng et al. [15] proposed to differentiate two distinct mechanobiological pathways for IAs growth and rupture, characterizing the complex pathophysiology involved. On the one hand, high WSS and positive wall shear stress gradient (WSSG) arising from fast flow impingement on the wall were found to enhance growth and rupture of small and thin-walled IA through mural-cell-mediated destructive remodeling. In this context, Blankena et al. [16] have shown an inverse correlation between wall shear stress and wall thickness. On the other hand, low WSS and high oscillatory shear index (OSI) arising in regions of slow and recirculating flow, were observed to promote the growth and rupture of large and thick-walled IAs through inflammatory-cell-mediated destructive remodeling.

Nowadays, phase contrast magnetic resonance imaging (PC-MRI) is widely used as a non-invasive and non-ionizing technique for in vivo blood flow measurements [7]. Nevertheless, this method was limited because of the inherent poor signal-to-noise ratio (SNR) of low-field MRI, which hindered the improvement of spatial resolution, and the long scan time of 4D PC-MRI [17].

In the last few years, a lot of effort has been put into quantifying WSS on the basis of experimentally measured velocity data, acquired with PC-MRI either in intracranial aneurysms [12], carotid arteries [18], femoral arteries [19] or aorta [20]. However, as reported by Potters et al. [20] and Petersson et al. [21], WSS evaluation with PC-MRI can be hampered by multiple factors such as segmentation inaccuracies, low spatial resolution, low SNR, and choice of VENC. In fact, several works [12, 20, 22] confirmed that WSS estimate increases with improved 4D PC-MRI spatial resolution. The recent introduction of ultra-high field (UHF) 7 T MRI, combined with the development of image acceleration techniques, might allow to overcome these limitations through an increase of SNR [23]. The gain in SNR can be exploited to enhance the spatiotemporal resolution and/or reduce the scan time. This feature then serves for a better visualization and quantification of hemodynamic parameters, particularly needed in small intracranial vessels. Various groups performed 7 T PC-MRI measurements to show the impact of field strength on aortic flows [24, 25]. Besides, in a pioneering study, van Ooij et al. [26] used 7 T PC-MRI to assess the hemodynamics inside intracranial arteries and proved that higher fields lead to better visualization and quantification of flow patterns in small vessels. Since then, few authors [16, 27–30] have carried out 7 T PC-MRI experiments to quantify flows inside IAs. The most recent work [30], based on a cohort of 5 patients harboring IAs, demonstrated that higher OSI values are reached with an increased temporal resolution, but did not find a clear similar relationship for WSS.

The purpose of the present study is to demonstrate how 7 T PC-MRI can provide a detailed visualization and characterization of near-wall hemodynamic parameters (WSS and OSI) inside in vitro models of patient-specific cerebral aneurysms.

## Material and methods

### Patient-specific aneurysm models

**Fig. 1:**
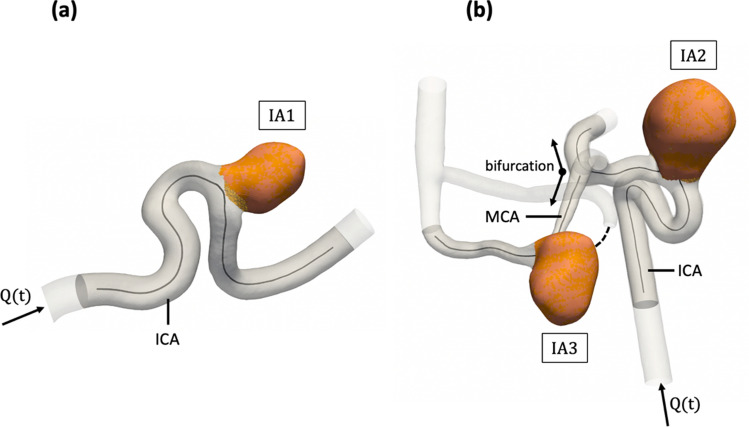
Patient-specific saccular aneurysms geometries obtained through segmentation of 3D TOF sequences’ images.** a** Carotid-ophtalmic aneurysm in model 1 (IA1), **b** carotid-ophtalmic aneurysm (IA2) and middle cerebral artery (IA3) in model 2. $\mathrm{Q}(\mathrm{t})$ refers to the inlet physiological flow rate. The measurement regions (PC-MRI sequence slices) are shown in opaque grey, and aneurysm sacs are highlighted in orange

The study was carried out on three saccular patient-specific IAs silicone models (see Fig. 1) based on two patient anatomies imaged with 3D rotational angiograms. The aneurysms were respectively located, for model 1: in the ophthalmic portion of the internal carotid artery (ICA) (IA1); for model 2: in the ophthalmic portion of the internal carotid artery (IA2) and in the middle cerebral artery (MCA) (IA3). Therefore, IA1 belonged to model 1 while IA2 and IA3 were both in model 2.

The geometrical characteristics of each aneurysm are given in Table 1.

**Table 1:** Geometrical characteristics of the three aneurysms

Aneurysm Id	IA1	IA2	IA3
Maximum height [mm]	8.57	13.5	10.04
Perpendicular height [mm]	8.31	12.93	6.99
Maximum width [mm]	8.61	14.61	8.88
Volume [${\mathrm{mm}}^{3}]$	314	1381	398
Surface [${\mathrm{mm}}^{2}]$	220	593	273
Inlet parent artery diameter [mm]	3.35	3.43	1.52
Neck diameter [mm]	6.72	9.55	11.62
Aspect ratio [−]	1.24	1.35	0.88

For the calculation of these parameters, the neck plane was defined as the best fitting plane (in a non-linear least square sense) to the neck points coming from the sac extraction. Besides, the aneurysm sacs were semi-automatically isolated by removing all mesh cells within a diameter ${\mathrm{D}}_{\mathrm{vessel}} + 1\mathrm{mm}$ around the center line [31].

### PC-MRI and 3D time-of-flight (TOF) sequence

All the measurements were performed on a 7 T MR system (Magnetom Terra, Siemens Erlangen, Germany) using a 8TX/32RX head coil (Nova Medical, USA). The 3D time-resolved (CINE) PC-MRI sequence consisted of three-directional velocity encoding and Compressed Sensing (CS) reconstruction to achieve higher acceleration rates [32, 33]. To select the optimal VENC in each model, we computed a 2D sequence for one slice positioned perpendicular to the main flow direction and located where we expected the highest velocities. We then checked that no or only a few voxels were aliased on the phase image to validate its value. The acquisition was performed using a prospective internal trigger, generated at the main physiological frequency (1 Hz). The acquisition parameters of both 3D TOF and PC-MRI sequences are summarized in Table 2.

**Table 2:** 7 T MRI 3D TOF and PC sequence parameters

Model Id	1 (IA1)		2 (IA2 and IA3)	
Sequence	TOF	PC	TOF	PC
TR/TE [$\mathrm{ms}]$	17/ 3.51	6.1/3.18	17/ 3.51	6.1/3.18
Flip angle [$^\circ ]$	24	12	24	12
FOV [${\mathrm{mm}}^{3}]$	156 $\times 146\times 44$	$112\times 112\times 40$	156 $\times 146\times 50$	$112\times 112\times 48$
Acquired Voxel [${\mathrm{mm}}^{3}]$	$0.3\times 0.3\times 0.3$	$0.5\times 0.5\times 0.5$	$0.3\times 0.3\times 0.3$	$0.5\times 0.5\times 0.5$
Temporal resolution [$\mathrm{ms}]$	–	$49$	–	$49$
Acquisition time [$\mathrm{min}]$	12:12	26:51	13:33	25:23
Phases	–	21		21
Acceleration	GRAPPA 2	CS 7.6	GRAPPA 2	CS 7.6
VENC [m/s]	–	1.1	–	1.2

### Fluid circulating system

The test bench was a closed circuit made of a controllable gear pump (MCP-Z-201, Ismatec) connected to a 5L tank. The blood analogue was a mixture of glycerine (59.4%) and water (40.6%), maintained at the physiological temperature of 37 °C with the help of an automated heater placed in the tank. The resulting fluid density $\uprho =1142\mathrm{ kg}/{\mathrm{m}}^{3}$ and kinematic viscosity $\upnu =4.67\cdot {10}^{-6}{\mathrm{ m}}^{2}/\mathrm{s}$ were similar to the ones of human blood at high shear rate [26, 27]. The generated flow rate had a typical ICA flow waveform [34] and was measured with an ultrasonic flowmeter (ME 6PXN, TS410, Transonic) located at the inlet of the model (see [35–37] for more details on the test bench).

**Fig. 2:**
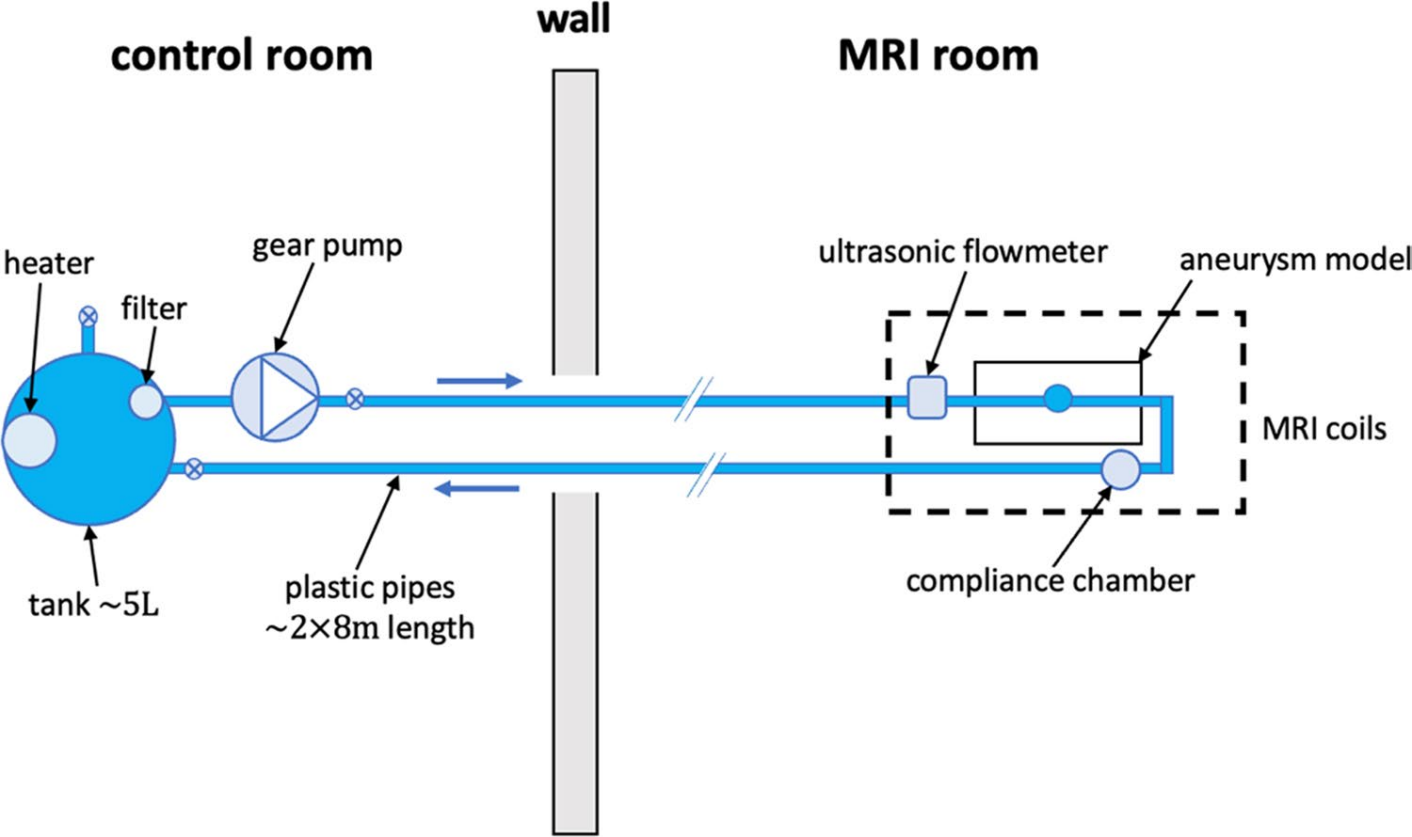
Schematic representation of the experimental setup in the MRI environment

To cope with the MRI requirements [29], we have placed the metallic components of the test bench outside the MRI room to avoid electromagnetic interferences. Therefore, we had to deploy a long plastic pipe of about 16 m length and 6 mm internal diameter to connect these components with the IA model, located in the MRI head coils (see Fig. 2). A special attention was paid to the issue of wave propagation within the long pipe and its reflection on the model, which might be responsible for a significant distortion of the flow rate in the model. This led to the design of a compliance chamber that drastically reduced this distortion when placed at the model outlet. Furthermore, additional boxes filled with a 2%wt water/agarose mixture were attached around the silicone models to increase the MR signal within the head coil and ensure a reliable background phase error correction [38].

### Data pre- and post-processing

The image reconstruction procedure included a background phase error correction to correct phase distortions arising from magnetic field inhomogeneity, eddy currents effects and Maxwell term [39].

An in-house temporal aliasing correction method [40] was used to remove the remaining $2\uppi$ phase jumps occurring around the systole. In fact, the phase was unwrapped by locally detecting the phase differences larger than +$\uppi$ or smaller than -$\uppi$ between two-time steps, using the diastolic velocity as the baseline. To accurately delineate the boundaries of the vessels, a 3D TOF sequence was first performed, which provided a higher spatial resolution compared to the PC sequence.

The vessel segmentation, based on 3D TOF images, was computed using geodesic active contour method implemented in ITK-SNAP software [41]. The surface was then smoothened using a low pass Taubin filter (vascular modeling toolkit, VMTK library) to reduce irregularities and discretization effects. Finally, the vessel centerline was again calculated in VMTK and served as reference for computing the orthogonal planes needed for flow rate assessment. Subsequently, a semi-automated rigid body co-registration was applied to the segmented vessel geometry to precisely delineate the fluid-circulating volume on the 3D PC-MRI data. The registration parameters (i.e. translation and rotation) were then optimized using a Quasi-Newton algorithm implemented in MATLAB (The Mathworks, Inc. Natick, USA) [40]. The remaining spurious vectors (i.e. outliers) found in the vicinity of the wall were corrected with a normalized median test [42] using 8 neighbor vectors and a detection threshold of 1. The 3D PC-MRI data (velocity field) and boundaries of the segmented vessel were interpolated with a spatial resolution of 0.1 mm onto measurement planes that were orthogonal to the centerline and equally spaced. All visualizations of the output parameters were carried out in ParaView (version 5.10.0; Kitware, Clifton Park, NY USA).

The SNR, which is a crucial quantitative indicator of an MRI system's performance, was calculated from the PC magnitude images following a “difference method” [43–45] that enabled estimation of the noise in the presence of the signal. The calculation was performed for each slice $\mathrm{k}$ according to [26, 46] using the PC magnitude signals $\mathrm{S}\left(\mathbf{r},{\mathrm{t}}_{\mathrm{i}}^{*},\mathrm{k}\right)$ and $\mathrm{S}\left(\mathbf{r},{\mathrm{t}}_{\mathrm{i}+1}^{*},\mathrm{k}\right)$ of two consecutive cardiac phases of similar mean velocity magnitudes (relative difference $\lesssim$ 1%). Since several pairs of cardiac phases met this criterion around diastole, we selected the one that showed the highest SNR. The chosen high signal region of interest (${\mathrm{ROI}}_{\mathrm{vessel}}$) corresponded to the vascular lumen. This SNR was then averaged over the number of slices ${\mathrm{N}}_{\mathrm{slices}}$ and took the following form:

1 $$\mathrm{SNR}=\underset{\begin{array}{c}k=1\dots {\mathrm{N}}_{\mathrm{slices}}\\ \end{array}}{\mathrm{mean}}\left[\frac{\underset{\begin{array}{c}r\in RO{\mathrm{I}}_{\mathrm{vessel}}\\ \end{array}}{\mathrm{mean}}\left(\mathrm{S}\left(\mathbf{r},{\mathrm{t}}_{\mathrm{i}}^{*},\mathrm{k}\right)+\mathrm{S}\left(\mathbf{r},{\mathrm{t}}_{\mathrm{i}+1}^{*},\mathrm{k}\right)\right)}{\sqrt{2}\underset{\begin{array}{c}r\in RO{\mathrm{I}}_{\mathrm{vessel}}\\ \end{array}}{\mathrm{stddev}}\left(\mathrm{S}\left(\mathbf{r},{\mathrm{t}}_{\mathrm{i}}^{*},\mathrm{k}\right)-\mathrm{S}\left(\mathbf{r},{\mathrm{t}}_{\mathrm{i}+1}^{*},\mathrm{k}\right)\right)}\right]$$

where $\mathbf{r}$ refers to the voxel position. Because the SNRs of the PC magnitude and phase images are proportional [46, 47], we did not estimate the SNR of the phase images separately. Moreover, the velocity-to-noise ratio (VNR) [48, 49] was calculated globally using the spatial average of the magnitude of the time-averaged velocity field as velocity signal:

2 $$\mathrm{VNR}=\frac{\uppi }{\sqrt{2}}\frac{\underset{\begin{array}{c}r\in V\end{array}}{\mathrm{mean}} \left|\overline{\mathbf{v} }\left(\mathbf{r}\right)\right|}{\mathrm{VENC}}\mathrm{SNR}$$

Here, V corresponds to the total volume of fluid comprising the parent artery and the aneurysm, and $\overline{\mathbf{v} }$ is the time-averaged velocity field.

### Flow rate measurement in the parent vessel

The accuracy of the method, which is a crucial step, was conducted through (i) a verification of the mass conservation (incompressible flow with rigid boundaries assumption) along the artery and (ii) a comparison of the flow rate with the one measured with the ultrasonic flowmeter. At each time step, the flow rate was evaluated from the velocity field at the selected planes along the centerline using a partial volume correction method [40].

### Wall shear stress and derived parameters on IAs sacs

The Cauchy stress tensor ${\varvec{\upsigma}}$ for a Newtonian fluid is defined as follows:

3 $${\varvec{\upsigma}}=-\mathrm{p}\mathbf{I}+{\varvec{\uptau}}$$

where $\mathrm{p}$ is the hydrostatic pressure, $\mathbf{I}$ is the identity matrix, ${\varvec{\uptau}}=2\upmu \mathbf{D}$ is the viscous stress tensor, $\upmu$ is the dynamic viscosity, $\mathbf{v}$ is the velocity field and $\mathbf{D}=1/2\left(\nabla \mathbf{v}+\nabla {\mathbf{v}}^{\mathrm{T}}\right)$ is the rate of deformation tensor. The wall shear stress was computed according to Crosetto et al. [50] using the algorithm from Potters et al. [20] implemented in MATLAB. Its expression in the local basis $\mathrm{B}=\left\{{\mathbf{t}}_{1},{\mathbf{t}}_{2},\mathbf{n}\right\}$ associated to each point on the wall is the following:

4 $$\mathbf{W}\mathbf{S}\mathbf{S}={\varvec{\upsigma}}\mathbf{n}-\left({\varvec{\upsigma}}\mathbf{n}\cdot \mathbf{n}\right)\mathbf{n}=\upmu {\left(\frac{\partial {\mathrm{v}}_{{\upxi }_{1}}}{\partial {\upxi }_{3}}, \frac{\partial {\mathrm{v}}_{{\upxi }_{2}}}{\partial {\upxi }_{3}},0\right)}_{\mathrm{B}}^{\mathrm{T}}$$

where ${\mathbf{t}}_{1},{\mathbf{t}}_{2}$ are the orthonormal vectors on the tangent plane, and $\mathbf{n}$ is the normal vector. Besides, $\left({\upxi }_{1},{\upxi }_{2},{\upxi }_{3}\right)$ is the coordinate system associated with basis $\mathrm{B}$. The tangential derivatives were set to zero, ${\partial }_{{\upxi }_{1}}{\mathrm{v}}_{{\upxi }_{3}}={\partial }_{{\upxi }_{2}}{\mathrm{v}}_{{\upxi }_{3}}=0$, due to the non-slip condition at the fixed wall. In the computation procedure, 4 interpolation points were taken along the normal direction (including the wall point ${\mathbf{v}}_{\mathrm{wall}}=0)$, the length of which was set to 1.5 mm. In this method, the inward wall normal vectors were estimated from the scattered points delineating the sac wall thanks to a principal component analysis (PCA) using the twenty nearest neighbor points. Moreover, the time-averaged WSS, defined over one cardiac cycle of period T, was computed as:

5 $$\mathbf{T}\mathbf{A}\mathbf{W}\mathbf{S}{\mathbf{S}}_{ }=\frac{1}{\mathrm{T}}\underset{0}{\overset{\mathrm{T}}{\int }}\mathbf{W}\mathbf{S}\mathbf{S}\mathrm{dt}$$

Finally, the oscillatory shear index [51, 52], which accounts for the directional variations of WSS during a cardiac cycle, was calculated as follows:

6 $$\mathrm{OSI}=\frac{1}{2}\left(1-\frac{\Vert \underset{0}{\overset{\mathrm{T}}{\int }}\mathbf{W}\mathbf{S}\mathbf{S}\mathrm{dt}\Vert }{\underset{0}{\overset{\mathrm{T}}{\int }}\Vert \mathbf{W}\mathbf{S}\mathbf{S}\Vert \mathrm{dt}}\right)\in \left[0-0.5\right]$$

## Results

### SNR and VNR

The SNR and VNR values for the three models are reported in the Table 3.

**Table 3:** SNR and VNR for each IA

Aneurysm Id	1	2	3
$\mathrm{SNR}$	95.61	154.59	100.81
$\mathrm{VNR}$	58.31	56.17	62.66

The average SNR and VNR over the three IA models were 117 and 59.05, respectively.

### Validation of PC-MRI flow measurements

**Fig. 3:**
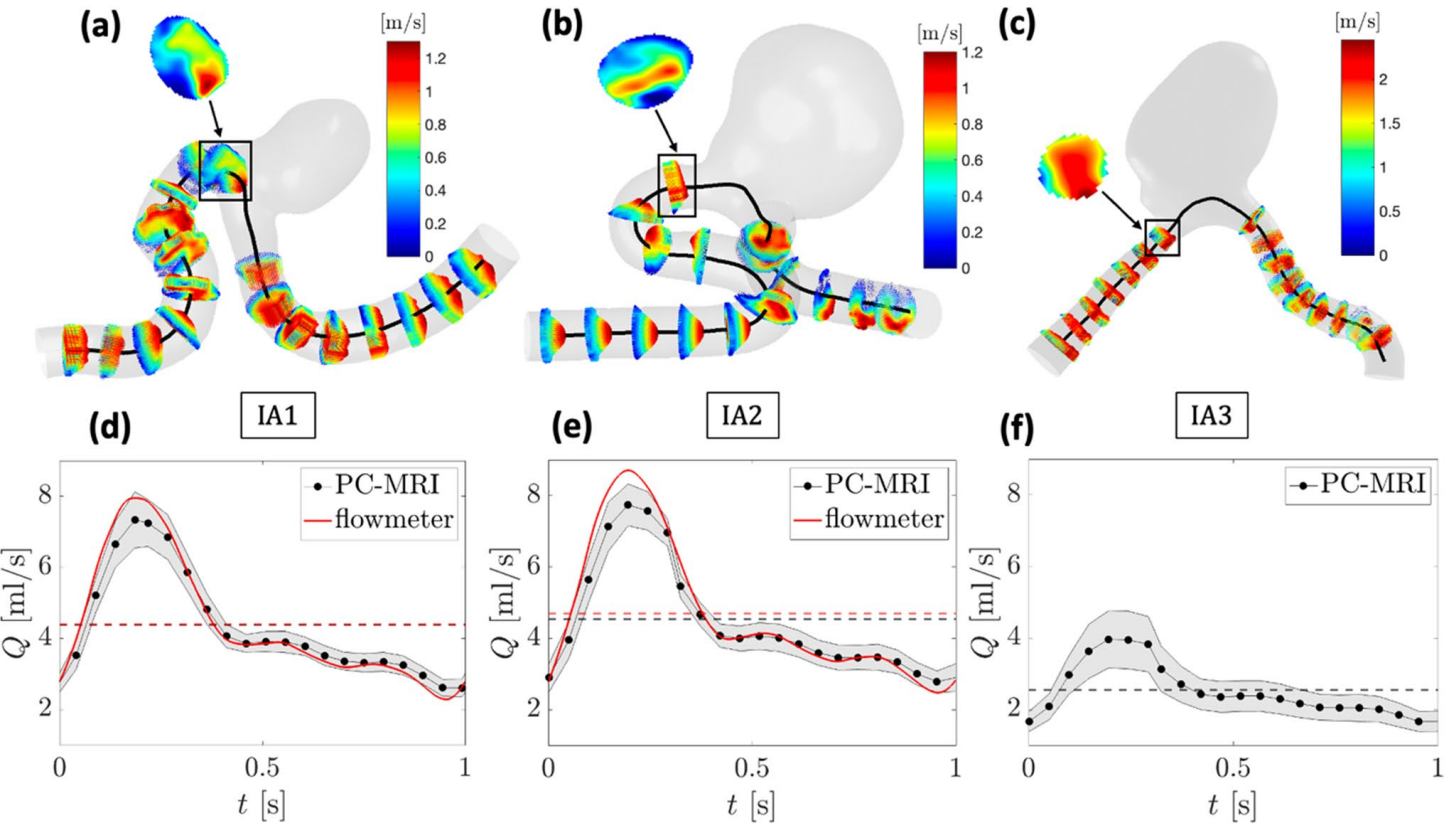
Systolic velocity profiles (normal component) on each measurement plane along the parent artery in **a** IA1, **b** IA2, **c** IA3. The velocity contours at the aneurysm inlet are highlighted (square box). Corresponding measured flow rates in **d** IA1, **e** IA2, **f** IA3. Red curve: flow rate measured from ultrasonic flowmeter. Black dots: inter-plane mean flow rate measured from PC-MRI. The part filled in grey represents the inter-plane flow rate standard deviation. Dashed lines: time-averaged flow rates

The pulsatile flow rates were computed across 16 to 18 normal planes along the parent artery by spatial integration of the normal component of velocity (see Fig. 3a–c) at each time step of the cardiac cycle. For each IA, the mean Reynolds number based on the time-averaged flow rate $\overline{\mathrm{Q} }$ (4.38, 4.53, 2.55 ml/s) and on the proximal IA diameter $\mathrm{D}$ (see Table 1) was ${\mathrm{Re}}_{\mathrm{m}}=4\overline{\mathrm{Q} }/\mathrm{\pi \nu D}=356, 360, 458,$ respectively. Moreover, the Womersley number derived from the main pulsation ${\upomega }_{0}=2\uppi {\mathrm{f}}_{0}$ (${\mathrm{f}}_{0}=1\mathrm{Hz})\mathrm{ was} \mathrm{\alpha }=\mathrm{D}/2\sqrt{{\upomega }_{0}/\upnu }=1.94$, 1.99, 0.88, respectively. Since these two dimensionless numbers were relatively “low”, we particularly observed a parabolic-like profile on the planes located in the upstream straight section of IA2 (see Fig. 3b).

The time evolution of the inter-plane mean and standard deviation flow rates measured in each IA with PC-MRI is plotted in Fig. 3d–f. The flow rates measured with the ultrasonic flowmeter are shown in the same figure for comparison. However, this comparison was not performed for IA3 because this aneurysm was located downstream of a vessel bifurcation in model 2 (see Fig. 1b) and was consequently not accessible with our flowmeter. For the same reason, the flow rates measured after the bifurcation in IA2 were not included in the inter-plane mean. In addition, the flow rate in the adjacent branch of IA3 was not calculated because we could not segment the thinner part (see dashed curve in Fig. 1b) due to the low TOF signal (diameter smaller than voxel size). Still, the velocity field within the aneurysm in.

**Fig. 4:**
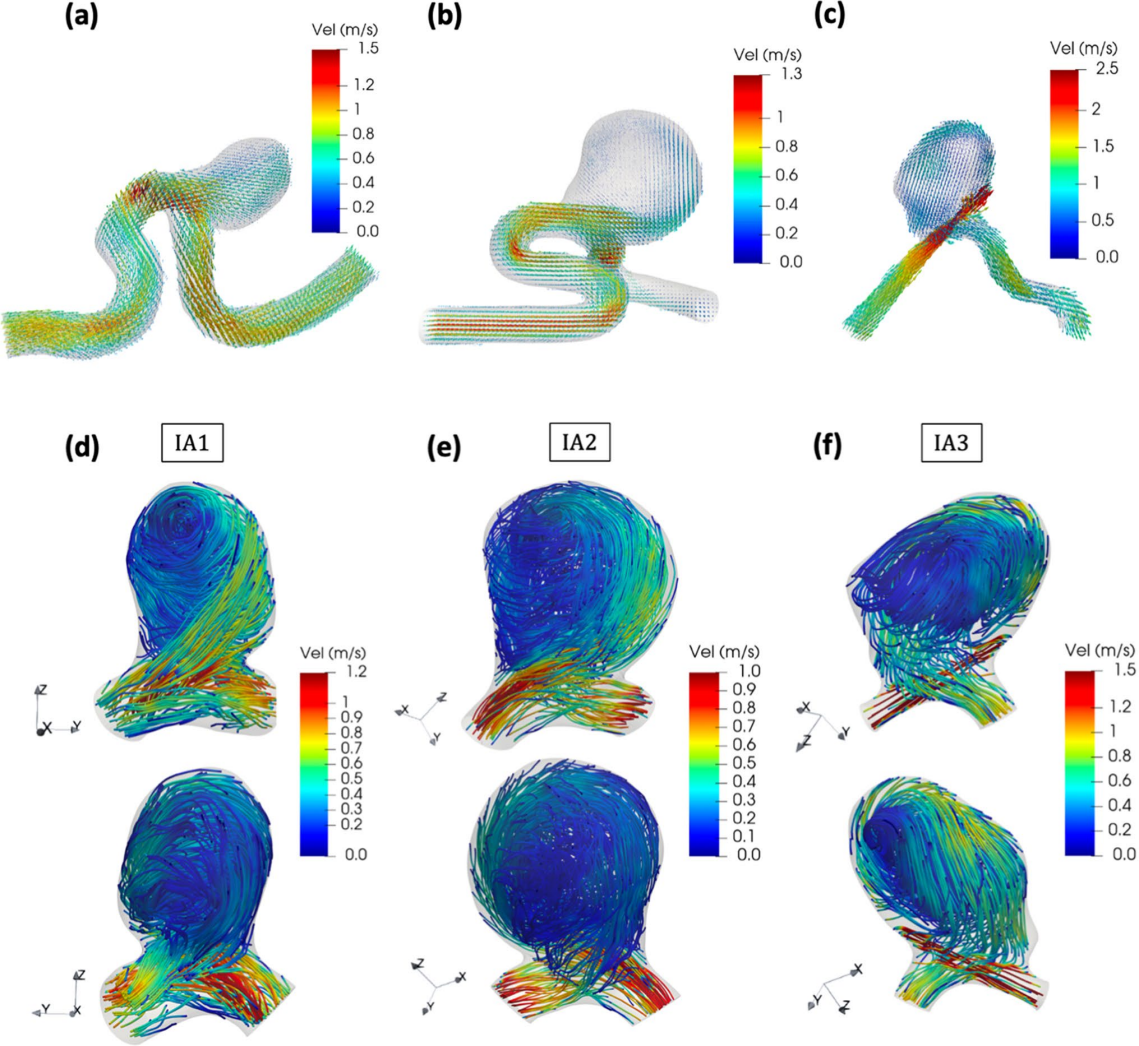
Overall systolic velocity field in **a** IA1, **b** IA2, **c** IA3. Streamlines inside the aneurysm sacs at systole in **d** IA1, **e** IA2, **f** IA3. Each IA is shown in two orientations

Figure 4c, f showed no flow entering this branch, confirming the validity of the omission of this region. It is worth underlining that the flow rate entering IA3, which depended on the relative hydraulic resistance of the two branches, was not specifically adjusted. Nevertheless, the time-averaged flow rate measured in the parent artery of IA3 ($\sim 2.5$ ml/s) was close to in vivo MCA flow rates [28], supporting its physiological relevance.

Figure 3d, e shows that the mean inter-plane flow rates measured with PC-MRI (IA1 and IA2) were in good agreement with the flow rates obtained with the ultrasonic flowmeter. However, we observed that the peak systole flow rate was slightly lower when computed from PC-MRI. This partly resulted from the usual temporal filtering of PC-MRI sequences, which smoothened the peak systole because of the limited temporal resolution ($\approx 20 \mathrm{Hz})$. The time-averaged difference was 4.2% at maximum while the largest systolic difference reached 11.1%, both in IA2. The highest inter-plane standard deviations were found in IA3, due to the smaller vessel diameter, which led to a more difficult segmentation and thus to an enhanced partial volume effect. For the other aneurysms, the maximum time-averaged inter-plane standard deviation was 9.4% in IA2, whereas the largest systolic inter-plane standard deviation reached 10.8% in IA1.

### Aneurysms flow patterns

Figure 4 shows the systolic velocity field and streamlines inside the aneurysm sacs. We focused on the systolic phase, where the velocities are the highest and the noisy data minimized, to get a clear visualization of the characteristic flow patterns. For each IA, the velocity magnitude was in average higher in the parent artery than in the aneurysm sac. The maximum velocity was almost the same for IA1 and IA2, which have similar parent artery diameters. In contrast, despite the lower flow rate, this maximum velocity almost doubled for IA3 as a result of the smaller upstream vessel diameter.

The streamlines first showed how the flow velocity varied along its trajectory inside the aneurysm. For IA3, the resulting jet-like flow impinged on the aneurysm wall and decelerated sharply as it initiated the swirl inside the sac. For IA1 and IA2, the impingement was less significant, resulting in a smoother velocity decrease. The characteristic velocity magnitudes encountered in each IA are given in Table 4. The streamlines also highlighted the main flow rotating structures. We indeed clearly identified the core of these vortical structures, which extended from one side to the other side of the sac and took the form of curved vortex tubes. Spotting out the time evolution of these vortices, we observed that their core lines slightly moved and deformed throughout the cardiac cycle.

**Table 4:** Characteristic velocity and WSS values for each IA

Aneurysm Id	1	2	3
$\underset{\mathbf{r}\in \mathrm{V}}{\mathrm{max}}\Vert \mathbf{v}(\mathbf{r},{\mathrm{t}}_{\mathrm{syst}})\Vert$[m/s]	0.94	0.92	2.53
$\underset{\mathbf{r}\in \mathrm{V}}{\mathrm{mean}}\Vert \mathbf{v}(\mathbf{r},{\mathrm{t}}_{\mathrm{syst}})\Vert$[m/s]	0.28	0.19	0.35
$\underset{\mathbf{r}\in \mathrm{V}}{\mathrm{mean}}\Vert \overline{\mathbf{v} }(\mathbf{r})\Vert$[m/s]	0.15	0.12	0.20
$\underset{\mathbf{r}\in \mathrm{S}}{\mathrm{max}}\Vert \mathbf{W}\mathbf{S}\mathbf{S}(\mathbf{r},{\mathrm{t}}_{\mathrm{syst}})\Vert$[Pa]	13.55	9.6	46.19
$\underset{\mathbf{r}\in \mathrm{S}}{\mathrm{mean}}\Vert \mathbf{W}\mathbf{S}\mathbf{S}(\mathbf{r},{\mathrm{t}}_{\mathrm{syst}})\Vert$[Pa]	5.24	3.15	7.6
$\underset{\mathbf{r}\in \mathrm{S}}{\mathrm{mean}}\Vert \mathbf{T}\mathbf{A}\mathbf{W}\mathbf{S}\mathbf{S}(\mathbf{r})\Vert$[Pa]	2.65	1.69	4.49

V corresponds to the aneurysm volume and S to the aneurysm surface, while $\mathbf{r}$ refers to the position in space. The index “$\mathrm{syst}$” relates to the systolic time step, while $\left(\stackrel{-}{}\right)$ denotes the time average

### WSS and its temporal variations

**Fig. 5:**
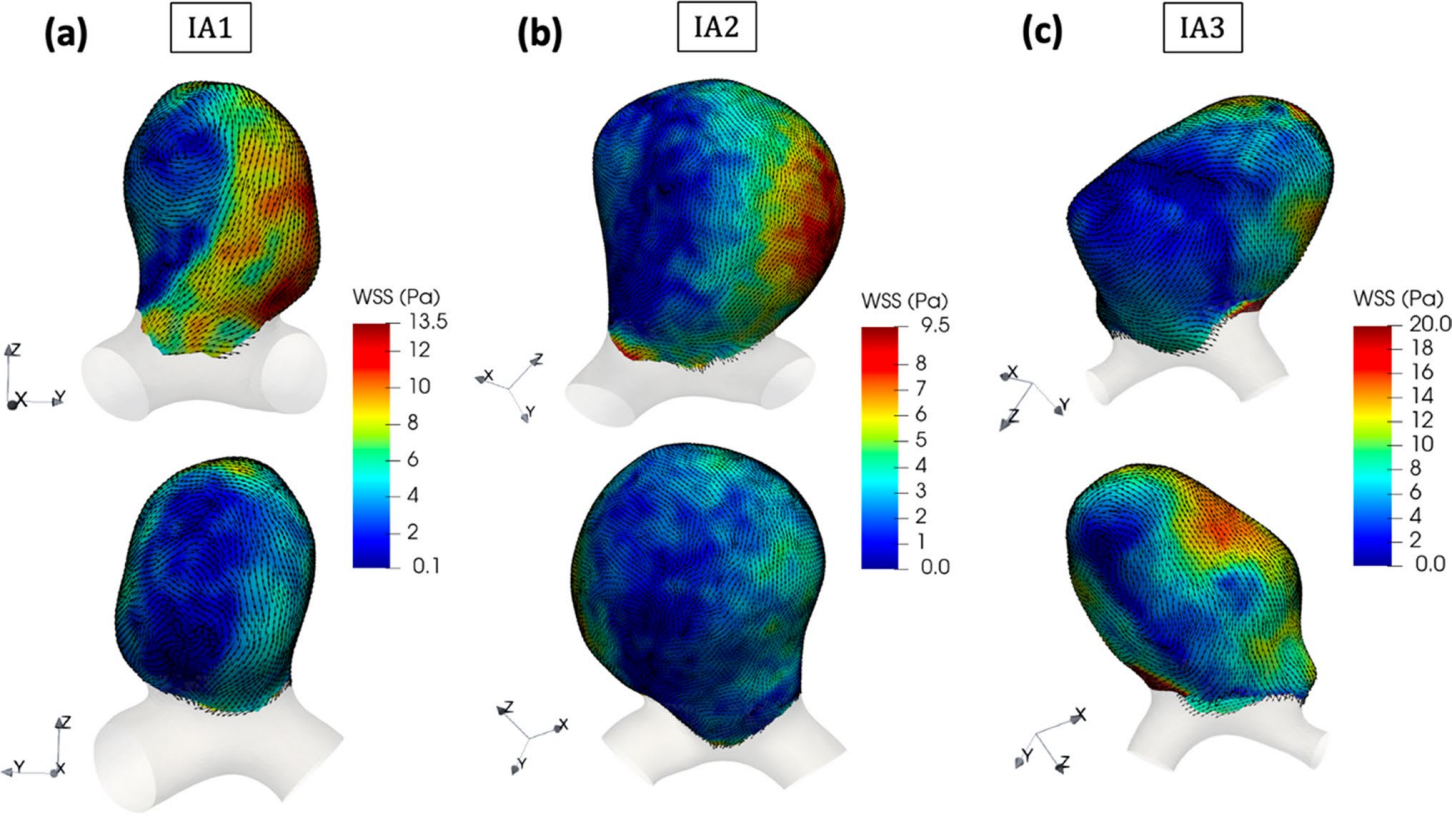
WSS magnitude at systole overlayed with unit vectors representing the direction of WSS in **a** IA1, **b** IA2, **c** IA3. Each IA is shown in two orientations

Because WSS is considered as a major physiological parameter for IA rupture assessment, we illustrate in Fig. 5 its distributions at systole for the three IAs considered. We again focused on the systolic phase to quantify the WSS maxima and enhance the contrast between high and low WSS regions. Noticeably, the WSS distribution was highly correlated with the direction and magnitude of the near wall-velocity field represented by the streamlines in Fig. 4d–f.

In each case, the maximum WSS occurred in the neighborhood of the impinging zone of the inflow jet on the wall, and its amplitude was mainly influenced by the magnitude and direction of the incident flow velocity. In fact, for IA3, the high inflow velocity $\sim 2.5$ m/s combined with the sharp incident angle led to a very concentrated and high WSS regions with a maximum around $\sim 46$ Pa. For IA1 and IA2, we observed a more diffused WSS maxima region and a smoother diminution throughout the vortical path. In contrast, the locations of the WSS minima corresponded to the tips of the large vortical structures and were characterized by higher directional fluctuations over time. Table 4 contains the characteristic WSS values found on the walls of each IA.

**Fig. 6:**
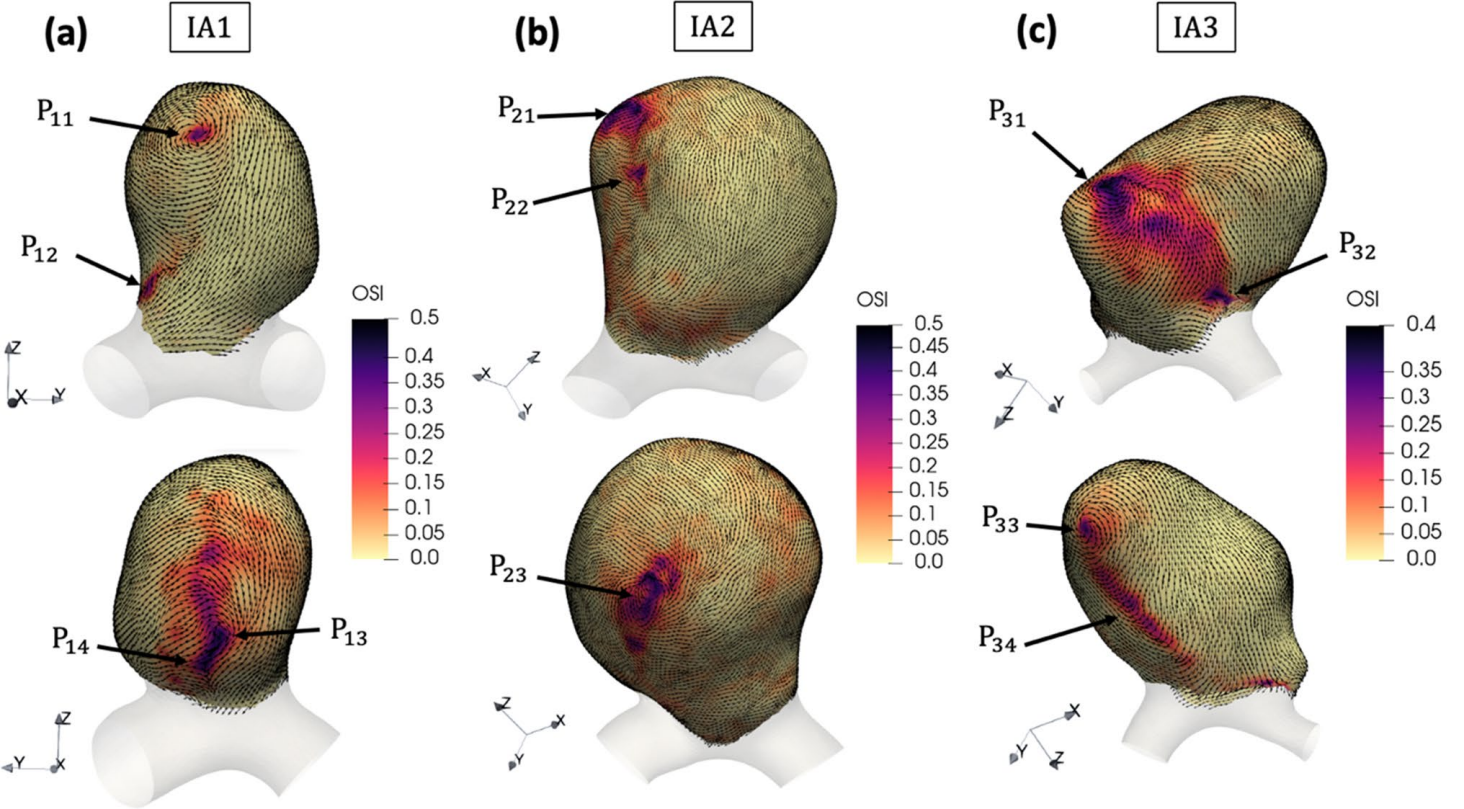
OSI overlayed with unit vectors representing the direction of time-averaged WSS in **a** IA1, **b** IA2, **c** IA3. Each IA is shown in two orientations. The different points ${\mathrm{P}}_{\mathrm{ij}}$ refer to specific regions of high OSI values

To account for the WSS temporal variations, we present the OSI distribution in Fig. 6 overlayed with the TAWSS vector field. The maximum OSI values were similar for all IAs and respectively equal to 0.47, 0.48 and 0.45. In all cases, the large OSI values were encountered at the locations where the TAWSS was the lowest. As illustrated in Fig. 6, these regions corresponded to either (i) the inner part of vortical structures or (ii) the intersection of flow streams coming from different directions. In the first case, the vortical structures matched the two tips of the large vortex found inside each IA sac. In the second case, the intersection of the streams coincided with the merge of the upward/downward flows at the vortex start/end.

These two WSS patterns are emphasized in Fig. 6, in which high OSI regions are pointed out by points ${\mathrm{P}}_{\mathrm{ij}}$ (i = aneurysm number, j = point number). The points corresponding to pattern (i) were located either in the center of small and well-defined vortical structures (${\mathrm{P}}_{11}$ and${\mathrm{P}}_{33}$) or inside large and fluctuating rotating flows (${\mathrm{P}}_{13}$,${\mathrm{P}}_{21}$,${\mathrm{P}}_{23}$,${\mathrm{P}}_{31}$). ${\mathrm{P}}_{12}, {\mathrm{P}}_{14}, {\mathrm{P}}_{22 }, {\mathrm{P}}_{32}$ and ${\mathrm{P}}_{34}$ refer to pattern (ii). It should be noticed that there were regions where these two patterns combined (e.g. ${\mathrm{P}}_{13}$ and${\mathrm{ P}}_{14}$), since they both developed in a close neighborhood.

## Discussion

In this study, we have presented quantitative measurements and a qualitative analysis of flow and WSS behavior in cerebral aneurysm models using 4D PC-MRI. The utilization of advanced image acceleration techniques in combination with 7 T MRI has resulted in an increase in SNR and an improvement in spatiotemporal resolution, all achievable within a reasonable acquisition time. This was demonstrated in one of the initial UHF studies on intracranial arteries by van Ooij et al. [26]. In our research, we integrated the accuracy of 7 T MRI acquisition with the post-processing methodology developed in prior works by Brina et al. [31] and Bouillot et al. [40, 53] to perform computations of velocity fields in the IAs. This allowed us to derive WSS and its temporal variations with improved accuracy.

We have shown that the gain in SNR and/or spatiotemporal resolution mainly enabled a detailed resolution of WSS patterns in two specific flow conditions. First, it allowed a precise visualization of WSS in low and noisy velocity regions through the gain of SNR. Second, the WSS estimation in high velocity gradient regions has been refined thanks to the enhanced spatial resolution.

Although the identification of high WSS magnitude regions is of great importance for the aneurysm rupture assessment, the gain in SNR and spatial resolution resulting from 7 T PC-MRI takes on its full meaning when characterizing low WSS patterns in regions of low and thus noisy velocity. Interestingly, we found a correspondence between regions of low WSS and sites of large WSS temporal variations characterized by an OSI above 0.2. In fact, regions of high OSI, were mainly found inside low WSS vortical structures at the tips of the vortex core line as well as at locations where two flow streams with different directions intersected. This statement was already reported by Cebral et al. [14] in a computational fluid dynamics (CFD) study on multiple IAs and by DiCarlo et al. [54], where they used stereo-PIV to deduce hemodynamic parameters in a stenosed carotid artery bifurcation. The only difference with the latter study is that, in our case, we did not identify elevated OSI values near flow impingement sites.

Besides, our results have demonstrated that the maximum WSS occurred around the flow impingement region on the aneurysm wall and that its magnitude was largely influenced by the velocity magnitude in the proximal parent artery. This observation follows the conclusions of the work of Meng et al. [15], in which it was claimed that flow acceleration adjacent to impingement points produces high WSS and positive WSSG.

These important WSS patterns, namely circular and elevated near impingement zones, were already observed with PC-MRI at 3 T by van Ooij et al. [12] for a large range of spatial resolutions going from 0.2 mm to 1 mm, but no correlation was made between low WSS vortical structures and high OSI values.

It is also important to notice that the identification of WSS fixed points (points where the WSS vector field vanishes) could help highlight regions of flow impingement, areas where the vortex structures interact with the wall, and locations of vector field intersection (flow separation) [55–57], which are closely associated with high OSI values. In fact, it is mathematically clear that the fixed points of the TAWSS take the maximum OSI value of 0.5 [58]. The classification of these fixed points is based on the eigenvalues of the Jacobian matrix of WSS in their vicinity and could serve to rigorously identify the locations of aneurysm rupture.

### Rupture risk assessment in clinical setting

The number of incidental findings of non-ruptured cerebral aneurysms has increased considerably in the last decade due to easier access to MRI in industrialized countries. The question of evaluating rupture risk versus treatment risk has become the subject of numerous hemodynamic research works, most notably numerical simulations [14, 59–61], but also with 4D PC-MRI [12, 40, 62, 63]. More recently, MR imaging of the aneurysm wall by suppression of the blood signal (black blood sequences) to detect wall enhancement after CA injection was reported as a biomarker of aneurysm instability [64, 65]. The ability to measure hemodynamic parameters at the wall level in the same acquisition modality, will provide complementary information about the wall morphology and will refine the risk of rupture. Local correlations of WSS and OSI with the vessel wall observations could also deliver relevant indications on the pathophysiology of growth and rupture of intracranial aneurysms, contributing substantially to the follow-up and management of such pathologies. Moreover, black blood sequences are prone to misinterpretation in regions of the aneurysm where velocity fields are slow or turbulent [64]. Coupling these sequences with accurate velocity field measurements, as proposed in our work, could help in carefully interpreting the vessel wall images by identifying sensitive areas.

However, despite an acceleration factor of 7.6 based on Compressed Sensing technique, the acquisition time of the PC-MRI sequences used in our study was still around 25 min, which is clinically impractical. For this experiment, the field of view was quite large, since it included a portion of parent artery before and after the aneurysm, in order to check the consistency of the PC-MRI measurements thanks to a flow rate validation at multiple vessel cross-sections (see Fig. 1). In combination with a smaller volume coverage in the slice direction (transversal orientation) and a reduction of the isotropic spatial resolution to about 0.75 mm, the scan time could be reduced to a clinically acceptable values of about 10 min. In addition, some 3 T PC-MRI studies have shown that the addition of a contrast agent (e.g. based on Gadolinium) to the fluid increases the SNR due to the shortening of T1 relaxation time [66, 67]. Here, to improve SNR, the imaging volume was positioned as perpendicular as possible to the flow direction to benefit from the inflow effect (thus using a relatively high flip angle regarding the high field strength of 7 T, see Table 2). Therefore, no contrast agent was used in our experiments since, in most cases, preparatory experiments did not reveal any further significant improvement in SNR with additional use of a Gadolinium based contrast agent (data not shown). However, for IA3, the slices could not be optimally positioned everywhere in the volume because multiple vessel branches spread in different directions. Thus, in that specific case, we encountered some difficulties in obtaining sufficient signal from TOF without contrast agent, especially in small vessels (see Fig. 1b).

### SNR and VNR benefits from ultra-high field

As depicted in Table 3, an elevated averaged SNR of 117 was found over the three tested IAs with the help of a “difference method”. Using the same method, van Ooij et al. [26] reported an averaged SNR of 31.7 in the cerebral arteries of five volunteers with 7 T PC-MRI at a spatial resolution of 0.5 mm. However, a straightforward SNR comparison with the present work is delicate because of the different acceleration techniques utilized, both of which have a specific impact on the measured SNR. On the one hand, they used SENSE parallel imaging with R = 3, which produces noise amplification characterized by the *g*-factor [68]. On the other hand, we used compressed sensing with R = 7.6 to reconstruct the images, a technique in which the SNR is strongly affected by the choice of regularization parameters. In fact, Sandino et al. [69] demonstrated that the perceived SNR can be improved by increasing the regularization parameters, but at the cost of blurring the images. Nevertheless, according to this paper, we could expect an increase of SNR by a factor of 2.6 when raising the field strength from 3 to 7 T which is, on average, larger than moving from to 1.5 to 3 T [24]. Since we used the same voxel size as well as multi-channel head coils in both studies, we would expect a similar increase in SNR compared to 3 T. This enhanced SNR can then serve to improve the spatial resolution, which in turn is beneficial for WSS computation.

Moreover, our single VENC acquisitions resulted in an average VNR of 59.05, which is high compared with other PC-MRI studies at lower fields [70, 71]. Unfortunately, no direct comparison was made with other 7 T PC-MRI works, because of the lack of similar data. It is interesting to mention that the VNR could have been improved by using a dual-VENC 4D flow sequence [72–74] at the expense of a longer scan time, or, by varying the VENC over the cardiac cycle [75] according to the time-dependent velocities. Such VNR improvement could be used to enhance the flow patterns in the low velocity regions, especially around diastole.

### Impact of field strength and sequence parameters on hemodynamic parameters

In the present case, apart from the VENC which varied according to the IA models, the sequence parameters were fixed for all experiments. However, numerous studies investigated the impact of the field strength and PC-MRI sequence type (spatiotemporal resolution, acceleration technique and factor) on near-wall hemodynamic parameters. First, Strecker et al. [76] compared systolic WSS at 1.5 T and 3 T but did not observe statistically different values. However, results from Wiesemann et al. [25] showed that WSS is significantly affected by field strength at 1.5, 3 and 7 T.

Second, Cibis et al. [22] demonstrated that OSI is dependent on both temporal and spatial resolution, while WSS is mostly dependent on spatial resolution. In the same trend, Gottwald et al. [30] showed that OSI increases with improved temporal resolution, but found no clear similar relationship for WSS in all aneurysms studied. Besides, in an in vitro IA study, van Ooij et al. [12] revealed that the spatially averaged WSS increases as the spatial resolution improves.

Third, Schnell et al. [77] observed that mean WSS obtained with *k-t* GRAPPA for acceleration factors R = 3, 5, 8 is significantly different from standard GRAPPA with R = 2 only when a 32-channel receiver coil is used. In addition, van Ooij et al. [46] demonstrated that SENSE acceleration produces more accurate results than *k-t* BLAST as a consequence of temporal blurring of the latter technique.

### In vitro measurement method

Compared to conventional optical velocity measurement techniques such as particle image velocimetry (PIV) or laser Doppler velocimetry (LDV), PC-MRI has the considerable advantage to work with opaque fluids like blood and to enable a straightforward computation of the 3D velocity field in complex vascular geometries. Moreover, it does not require any tracer particles that can alter fluid behavior. However, the major disadvantages compared to PIV are (i) a lower spatiotemporal resolution [78] with temporal filtering and (ii) the inability to perform statistics over multiple cardiac cycles [54], due to the already long scan time of the flow MRI sequences.

### Limitations and improvements

1. A limitation of the present study is the choice of a unique VENC, which must cover a large range of velocities in the aneurysm domain. This could lead to noisy velocity measurements in the near-wall low velocity regions. In addition, the limited temporal resolution due to the three-dimensional velocity encoding can filter the velocity values. Therefore, the high OSI values found in the low WSS regions could possibly be induced by noisy velocities (low SNR), which consequently represent unphysical temporal variations of WSS.
2. Moreover, even if the UHF improves the data quality, the WSS derivation depends on the PC-MRI spatial resolution, on the segmentation accuracy, and on several other parameters used in the WSS computation algorithm (length of the normal direction, number of points taken in this normal, fitting technique…).
3. This study was limited to three different aneurysms, which do not represent the full variability of geometries and hemodynamics [11] found in this pathology. However, the three geometries studied included two sidewalls (IA1 and IA2 are carotid ophthalmic aneurysms of different sizes) and one bifurcation (IA3 is a middle cerebral artery aneurysm), each of which exhibited different flow patterns.
4. The goal of our experiment was to faithfully reproduce a typical physiological intracranial flow rate inside our models to precisely quantify the near-wall hemodynamic parameters inside the IA sac. However, a relevant improvement could be to use a more advanced blood analogue that considers the non-Newtonian shear thinning nature of blood. In fact, the non-Newtonian behavior cannot be neglected in situations where the shear rate is low (typically under 1 ${0\mathrm{s}}^{-1}$ [79]) such as pulsatile flows conditions [80], small vessels [81] and regions of arterial geometrical changes [82]. Indeed, various authors [54, 83, 84] used a solution of water, glycerine and xanthan gum in PIV experiments to better mimic these rheological features. For the time being, no PC-MRI flow study has been performed inside IAs models with this type of working fluid.

Finally, to realize the transition between in vitro and clinical measurements, further technical improvements are needed to be applied in clinical routines. This could be achieved mainly by further shortening the acquisition time, for example, by restricting the region of interest to the neighborhood of the aneurysm sac. In this work, we took advantage of the higher spatial resolution of the TOF sequence to segment the arteries on the PC images. However, this geometrical co-registration adds computational time and can lead to misalignment issues, especially in very small and tortuous vessels where the signal from TOF is not sufficient. Therefore, the use of PC magnitude to perform this task should be of great interest if the spatial resolution can be lowered to around 0.3–0.4 mm. Additionally, the entire post-processing pipeline should be automated and integrated to facilitate the procedure for rapid diagnosis.

## Conclusion

We performed 7 T PC-MRI in in vitro experiments to evaluate the near-wall hemodynamic parameters in three patient-specific IAs. We developed an MRI-compatible test bench, which faithfully reproduced a typical physiological intracranial flow rate. We took advantage of recent developments of MR image acceleration techniques to derive highly resolved velocity field in a short scan time. Our results clearly demonstrate the remarkable ability of 7 T PC-MRI in identifying relevant WSS patterns, namely high WSS regions and low WSS vortical structures. We strongly believe that our study will pave the way for a better understanding of the role of WSS in IAs rupture, through systematic 7 T PC-MRI experimentations in a large cohort of patients.

## Data Availability

The data that support the findings of this study are available on the request of corresponding author AS.
